# Efficacy of various surgical approaches in treating hematospermia using transurethral seminal vesiculoscopy

**DOI:** 10.1186/s12893-023-02290-2

**Published:** 2023-12-21

**Authors:** Rui-Jie Yao, Hong Xiao, Shu-Shen Chen, Zhi-Hao Feng, Yi-Lang Ding, Xi Chen, Song-Xi Tang, Hui-Liang Zhou

**Affiliations:** Department of Andrology, The First Affiliated Hospital of Fujian Medical University, 20 Chazhong Road, Fuzhou, 350005 China

**Keywords:** Efficacy, Hematospermia, Natural orifice, Seminal vesiculoscopy

## Abstract

**Purpose:**

To explore the efficacy of different approaches of seminal vesiculoscopy surgery and the predictive factors of good treatment outcome.

**Materials and Methods:**

A retrospective analysis of 68 patients who underwent seminal vesiculoscopy for hematospermia in our hospital from January 2015 to January 2021. According to different surgical approaches, they were divided into three groups: natural ejaculatory ducts (method A, 45 cases), assisted transurethral resection/incision of ejaculatory ducts (method B, 14 cases), fenestration in prostatic utricle (method C, 9 cases). We analyzed the recurrence rate of the three surgical approaches and the predictive factors of treatment efficacy.

**Results:**

The total recurrence rate after the seminal vesiculoscopy for hematospermia in this group was 32.35%. The postoperative recurrence rates of the three methods were 24.44% for method A, 50.00% for method B and 44.44% for method C, and there was no significant difference among the three methods (*P* > 0.05). The data of five predictors of 45 cases in method A group were included in the Univariate Logistic analysis, the results suggest that whether complicated with seminal tract stones/cysts was an effective predictor (OR 0.250, *P* = 0.022), which was still an effective predictor in the Multivariate Logistic analysis model (OR 0.244, *P* = 0.010).

**Conclusions:**

The Transurethral seminal vesiculoscopy technique demonstrates a low postoperative recurrence rate in treating hematospermia. Among the various approaches, the intraoperative use of natural orifices through the ejaculatory duct exhibits the lowest recurrence rate. Additionally, seminal tract stones/cysts effectively predict favorable postoperative outcomes.

## Introduction

Hematospermia is a distressing symptom with various potential causes, including genitourinary system infections, vascular malformations, stones, inflammations, tumors, and systemic diseases associated with bleeding risks. While most cases are self-limiting, patients experiencing frequent or persistent hematospermia necessitate a thorough evaluation of urologist intervention [1].

Hematospermia treatment involves conservative and surgical approaches. Conservative treatment includes antibiotics against pathogenic microbial infections and medications like Finasteride for hematospermia caused by Benign Prostatic Hyperplasia. After excluding other organic diseases, Finasteride also can benefit patients with idiopathic and refractory hematospermia [2]. However, long-term medication and high recurrence rates are significant concerns with refractory hematospermia [3]. Surgical treatment includes open surgery, laparoscopic techniques, and transurethral seminal vesiculoscopy. Accessing the seminal vesicle can be challenging due to its deep location in the pelvis. Laparoscopy and open surgery require extensive dissection, are time-consuming, and carry risks, including severe bleeding and damage to peritoneal organs like the rectum [4]. In 1998, Yang et al. successfully performed an endoscopic examination of the seminal vesicles for the first time [3], opening new avenues for seminal tract and vesicle examination and treatment. Numerous clinical studies have shown the safety and efficacy of transurethral seminal vesiculoscopy [3]. According to the local patency of the ejaculatory duct orifice, the surgical approaches include the following: (A) the natural ejaculatory duct; (B) transurethral resection/incision of ejaculatory duct (TURED/TUIED) combined with seminal vesiculoscopy; (C) the fenestration in prostatic utricle (PU) combined with seminal vesiculoscopy; and (D) seminal vesiculoscopy through the pathological opening in PU [5]. Currently, there is limited comparative data available on the efficacy of different surgical approaches for treating hematospermia. Perhaps surgical procedures that can be performed through natural orifices without incisions preserve the original anatomical structure to a greater extent, and minimize local injury.Therefore, this study aimed to explore the efficacy of different surgical approaches for treating hematospermia using seminal vesiculoscopy.

## Materials and methods

### Clinical data

Between January 2015 and January 2021, data were collected from patients with hematospermiawho underwent seminal vesiculoscopy at the First Affiliated Hospital of Fujian Medical University. The Ethics committee of the same hospital approved the study. All patients failed or responded poorly to at least one conservative therapy for three months. Patients underwent routine Magnetic Resonance Imaging (MRI) scans of the seminal vesicle, including enhanced examination before surgery. Subsequently, all patients underwent seminal vesiculoscopy surgery.

Inclusion criteria for surgery were as follows: (1) Persistent, typical hematospermia; (2) Normal urine routine and prostatic fluid examination; and (3) Persistent or recurrent hematospermia despite 3–6 months of systemic antibiotics or local physical therapy.

Exclusion criteria were: (1) Patients with congenital abnormalities or genitourinary tract tumors confirmed by preoperative MRI and transrectal ultrasonography; and (2) Patients with systemic hemorrhagic disease.

The study included 68 patients aged 22–65 years, with an average age of 40.49 years. The disease duration ranged from 3–240 months, with a median of 14.5 months. Among the cases, 39 (57.35%) were unilateral, and 29 (42.65%) were bilateral. MRI scan revealed fresh bleeding in seven (10.29%) cases. Additionally, 43 (63.24%) cases had complicated with seminal tract stones/cysts. Method A was used in 45 (66.18%) cases, method B in 14 (20.59%) cases, and method C in nine (13.23%) cases (Table 1).

**Table 1:** Demographic data of enrolled patients

Age, year (mean ± SD)	40.49 ± 11.40
Duration, month, median (interquartile range)	14.50 (9.25, 36.0)
Side of the lesion, n (percentage)	
Unilateral	39 (57.35%)
Bilateral	29 (42.65%)
Fresh bleeding revealed by MRI, n (percentage)	
Yes	7 (10.29%)
No	61 (89.71%)
Seminal tract stones/cysts, n (percentage)	
Yes	43 (63.24%)
No	25 (36.76%)
Surgical approach, n (percentage)	
A	45 (66.18%)
B	14 (20.59%)
C	9 (13.23%)

A: the natural ejaculatory duct approach, B: transurethral resection/incision of ejaculatory duct (TURED/TUIED) combined with seminal vesiculoscopy, C: the fenestration in prostatic utricle (PU) combined with seminal vesiculoscopy

### Observation indicators

The variables analyzed in this study included age, disease duration, unilateral/bilateral lesions, seminal vesicle MRI showing fresh or non-fresh bleeding, whether complicated with seminal tract stones/cysts, and the surgical approach. Recurrence was assessed during a 1-year follow-up, with criteria for recurrence defined as first ejaculation in the first week of surgery, regular weekly ejaculations, and at least one ejaculation; the presence of visible blood in the semen one month after surgery was normal. It is a recurrence when visible blood remains in the semen three months after the operation. The predictive factors associated with postoperative outcomes were included in the statistical analysis.

### Surgical methods

Following anesthesia, the patient was placed in lithotomy position. The outer urethra was identified as the reference point, followed by routine disinfection and drape the area. A 12F catheter was inserted for drainage, and a Wolf 4.5/6.5F rigid ureteroscope was used as the surgical endoscope, connected to a TV imaging system. The endoscope was carefully introduced into the bladder through the urethra, using normal saline as the perfusion solution. The urethra and bladder were meticulously examined for stones, diverticulum, and tumors.

**Fig. 1:**
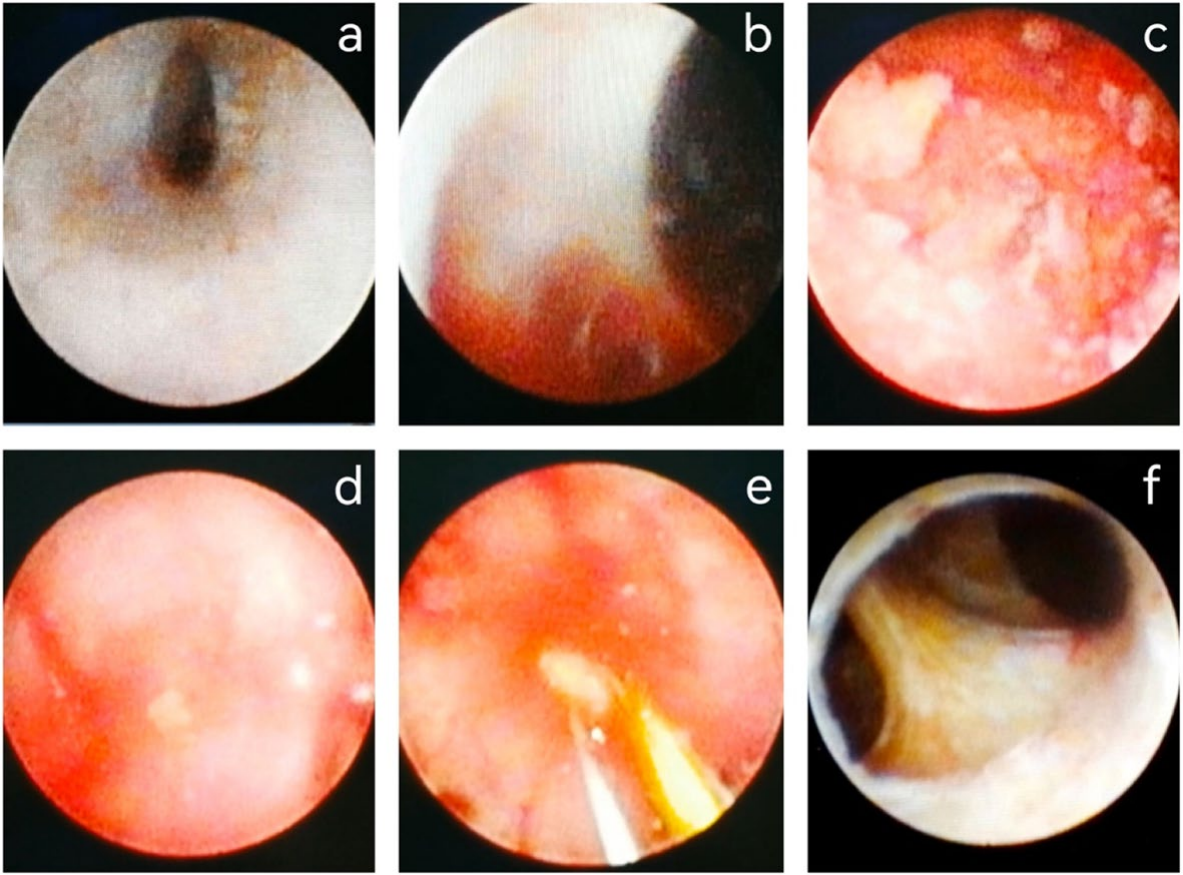
(Method A): the natural ejaculatory duct. After entered the cavity of the seminal vesicle, mixture which contains old blood clots and stones could be seen. Then we removed stones and rinsed with normal saline repeatedly

**Fig. 2:**
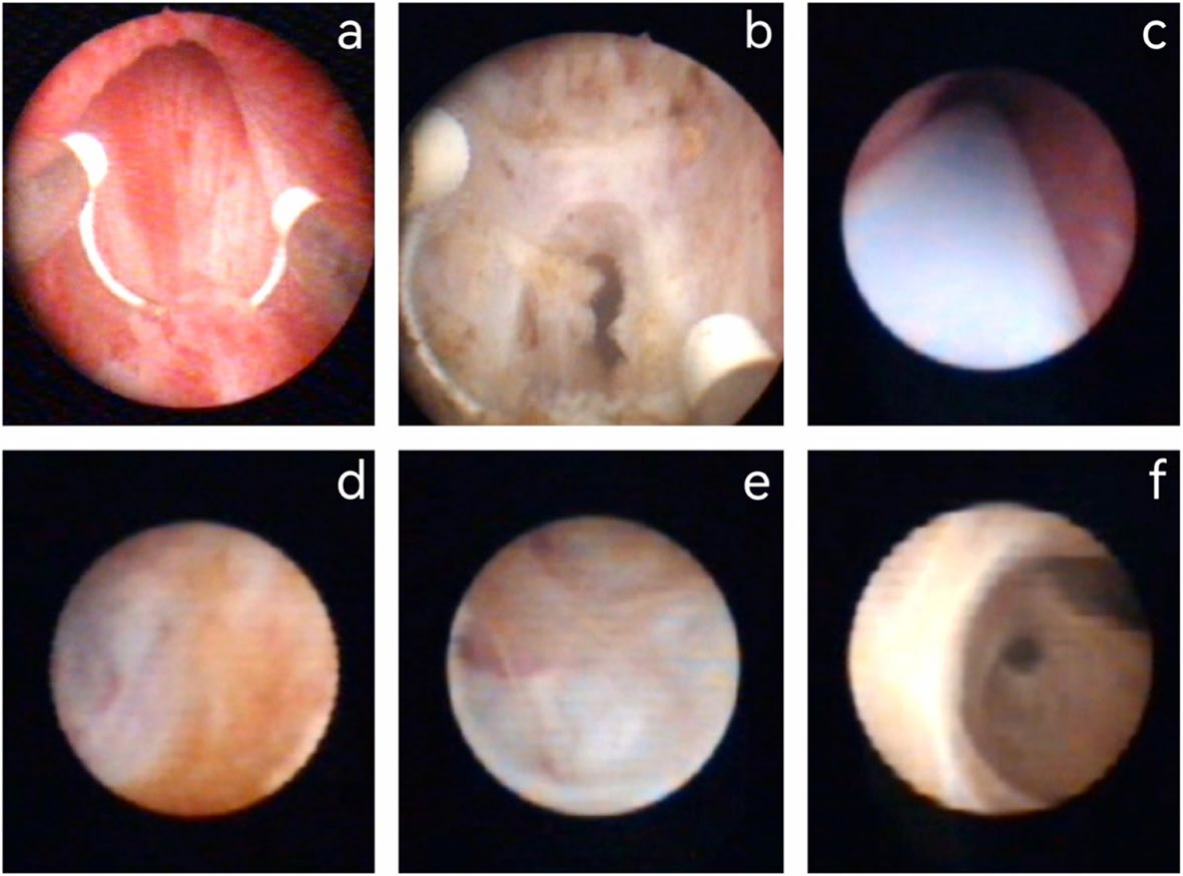
(Method B): Transurethral resection/incision of ejaculatory duct (TURED/TUIED) combined with seminal vesiculoscopy. Due to the obstruction of PU’s orifice, incision was performed through the ejaculatory duct of the urethra then into the seminal vesicle cavity

**Fig. 3:**
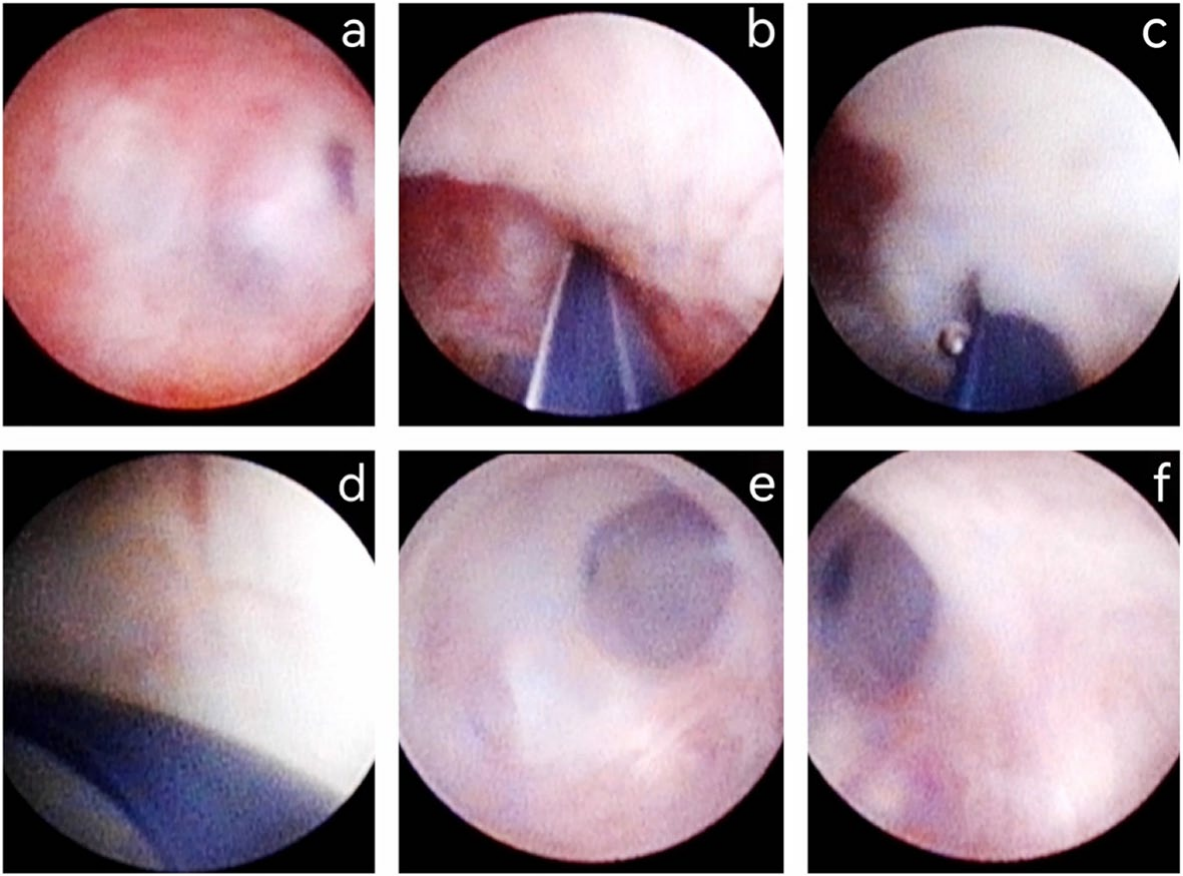
(Method C): The fenestration in prostatic utricle (PU) combined with seminal vesiculoscopy. Under the guidance of Zebra Urological Guidewire, we entered the seminal vesicle cavity through the side channel

Subsequently, the selection of the surgical approach was primarily based on exploring the ejaculatory duct orifice. Following are the detailed descriptions of the surgical procedures for each approach:

- Method A: Retract the endoscope body to the verumontanum to assess the normal appearance and shape of the verumontanum. Subsequently, the seminal vesiculoscope was inserted into the PU to explore and exit the PU. At the 5 o'clock position, the left seminal vesicle cavity was assessed to examine for bleeding, stones, cysts, or old blood clots. Normal saline was used for repeated rinsing. If stones were present, seminal vesicle lithotripsy was performed using a holmium laser to crush the stones into powder, followed by thorough rinsing and clearance. In cases of a seminal vesicle cyst or ejaculatory duct cyst, a relevant cystectomy was performed. The endoscope was then withdrawn to the left ejaculatory duct orifice, repositioning the PU, and subsequently, the right seminal vesicle was accessed at the 7 o'clock position in the PU. The exploration and treatment of the seminal vesicle cavity followed the same procedure (Fig. 1).

- Method B: Resection/incision through the ejaculatory duct of the urethra if the ejaculatory duct orifice or PU orifice were obstructed. Subsequently, the seminal vesicle cavity was accessed through the ejaculatory duct using the same approach; The exploration and treatment of the seminal vesicle cavity followed the same procedure (Fig. 2).

- Method C: If locating the ejaculatory duct orifice was challenging or when the orifice was obstructed or not amenable to recanalization at the PU, the seminal vesicle cavity was penetrated through the weak mucosa side channel, guided by the Zebra Urological Guidewire (Fig. 3).

During the operation, a routine bacterial culture of the seminal vesicle flushing fluid is performed for all patients. No active bleeding in the surgical cavity was confirmed. Following the procedure, a 16F three-chamber silicone urinary catheter was indwelled, and 15 ml of water was injected to complete the operation.

### Statistical analysis

The statistical analysis of the data was conducted using SPSS 26.0 software. Normally distributed data were expressed as mean ± standard deviation (x̅ ± S), whilenon-normally distributed variables were expressed as median (25% and 75% interquartile range). Categorical variables were presented with number (percentage).The postoperative recurrence rates for different methods were assessed using Fisher's test. The potential predictive factors were selected for Univariate Logistic analysis, and the factors with significant differences were further analyzed for Multivariate Logistic regression analysis to identify the predictive factors with good therapeutic effect. If p remained below 0.05, the predictors were considered statistically significant. Statistical significance was determined at *p* < 0.05 for bilateral analysis.

## Results

### Surgical efficacy

All 68 patients underwent the operation without obvious complications, including bleeding, perineal pain and discomfort, ejaculation dysfunction, orchitis, epididymitis, and others. Within one year after surgery, recurrence was observed in 11 patients (24.44%) treated with method A, seven patients (50.00%) treated with method B, and four patients (44.44%) treated with method C. No significant difference was observed in the recurrence rates of different approaches (*p* = 0.144). (Table 2).

**Table 2:** Comparison of the efficacy of three surgical approaches (*n* = 68)

Surgical methods	recurrent	Non-recurrent	Recurrence rate (%)	*P*
Method A	11	34	24.44	0.144
Method B	7	7	50.00	
Method C	4	5	44.44	

A: the natural ejaculatory duct approach, B: transurethral resection/incision of ejaculatory duct (TURED/TUIED) combined with seminal vesiculoscopy, C: the fenestration in prostatic utricle (PU) combined with seminal vesiculoscopy

Patients were informed before the operation that in certain cases of resistant hematospermia, the underlying cause of the condition may remain unclear even after surgery. In such instances, continuing conservative treatment can be considered if surgical treatment fails.

### Analysis of predictive factors of good postoperative outcome (A method)

Based on the one-year postoperative outcomes of hematospermia, five factors were analyzed, including age, disease duration, presence of stones/cysts in the seminal tract during surgery, unilateral/bilateral lesions, and the presence of fresh or non-fresh bleeding on seminal vesicle MRIs. A Multivariate Logistic analysis was performed specifically for method A due to a limited number of cases for the B and C methods. The analysis revealed that intraoperative complicated with seminal tract stones/cysts were statistically significant (*p* = 0.022 OR 0.250). Subsequently, multiple logistic analysis was conducted, and the significance remained (*p* = 0.010 OR 0.244). No significant logistic regression correlation was found between other factors and favorable surgical outcomes (*p* > 0.05). (Table 3).

**Table 3:** Logistic analysis of predictors of postoperative efficacy (*n* = 45)

	Univariate Logistic Analysis		Multivariate Logistic Analysis	
	*OR* (95%CI)	*P*	*OR* (95%CI)	*P*
Age	1.032 (0.981, 1.085)	0.220		
Duration of disease	1.008 (0.997, 1.019)	0.165		
Unilateral/Bilateral lesions	0.582 (0.173, 1.954)	0.381		
Whether combined with seminal tract stones/cysts	0.250 (0.076, 0.821)	0.022^*^	0.244 (0.083, 0.716)	0.010^*^
Whether the seminal vesicle MRI manifested fresh bleeding or non-fresh bleeding	0.281 (0.026, 3.102)	0.300		

*OR* Odds ratio, *CI* Confidence interval, * P < 0.05

## Discussion

Advancements in endoscopy and minimally invasive technology have revolutionized the management of diseases that traditionally require open surgery or pose challenges in treatment [6]. While hematospermia may not have serious consequences for men, its recurrent occurrence negatively impacts both male reproductive health and mental well-being and strains relationships between partners. Recently, seminal vesiculoscopy has emerged as a vital technique for addressing hematospermia, ejaculatory duct obstructive azoospermia [7], and other related conditions, gradually gaining popularity. This advancement has significantly benefited affected patients but has also raised the bar for urologists, demanding higher standards of practice. This study explored and compared the effectiveness of various surgical approaches and disease conditions in treating hematospermia using seminal vesiculoscopy.

### Current status of hematospermia and operation methods

Hematospermia refers to the presence of blood during ejaculation [8]. While generally benign and self-limiting, it often causes significant anxiety and distress in affected individuals. The condition is commonly associated with inflammation, infection [9, 10], tumors, or systemic diseases [11]. In many hematospermia patients, ejaculatory duct stones have been found to be the root cause of hematospermia [12]. If hemotospermia is accompanied by seminal vesicle or ejaculatory duct stones, preoperative imaging is very crucial. Computerized Tomography (CT) is unsuitable for detecting small and relatively soft seminal vesicle stones, which are often the cause of refractory hematospermia. Contrarily, MRI is a preferred modality for determining the location of blood accumulation within the seminal vesicle and detecting low signal shadows indicative of stones [13]. Transrectal ultrasound screening (TRUS) is also employed for evaluating patients and identifying ejaculatory duct stones in hematospermia patients [14–18]. However, MRI examination demonstrates a higher positive rate and better ability to detect stones primarily composed of protein materials, compared to TRUS [19–22]. Therefore, our research performed routine seminal vesicle MRI scans and enhanced examinations before surgery. Currently, the treatment options for hematospermia primarily include conservative and surgical approaches. Surgical treatments encompass open and laparoscopic surgery and transurethral seminal vesiculoscopy. Traditional open surgical approaches for treating primary or secondary lesions of the seminal vesicle glands have inherent drawbacks and necessitate significant incisions for access. These open surgeries involve extensive anatomical manipulation, leading to complications associated with exposed surgical fields, prolonged operative times, and intra-abdominal ruptures. To overcome these complications, laparoscopic and endoscopic methods are viable alternatives to conventional surgical interventions [23, 24]. Laparoscopy is the preferred surgical approach for large stones in the seminal vesicles or ejaculatory ducts [24]. Similarly, the seminal vesiculoscopy technique offers distinct advantages in the direct visualization and exploration of the distal end of the seminal tract, enabling accurate diagnosis and treatment of hematospermia. This technique involves the removal of blood clots, stones, and infected seminal vesicle fluid from the seminal vesicle cavity [4, 10]. Hence, seminal vesiculoscopy is highly recommended in the current clinical setting. Liao et al. [25] conducted a meta-analysis involving 584 patients and demonstrated that transurethral seminal vesiculoscopy was more effective than conventional drug therapy in treating hematospermia, with a lower recurrence rate. Compared to seminal vesicle puncture and catheterization, seminal vesiculoscopy shows comparable efficacy but lower rates of postoperative complications and hematospermia recurrence [17, 18, 26]. Wang et al. [27] reported that in 64 cases of seminal vesiculitis treated with seminal vesiculoscopy, complete resolution of hematospermia was observed in 52 cases, partial improvement in eight cases, and no change in four cases three months after the procedure. These findings confirmed the efficacy of seminal vesiculoscopy in treating hematospermic seminal vesiculitis, allowing for seminal vesicle inspection, removal of stones and inflammatory substances, and a lack of significant complications, making it a valuable clinical approach deserving of wider adoption. Our data revealed a total recurrence rate of 32.35% in hematospermia patients after seminal vesicle endoscopy, indicating a favorable efficacy of seminal vesiculoscopy in treating hematospermia. However, it is noteworthy that the operative space within the seminal vesicles is limited, requiring surgeons to be proficient in systematic intracavitary techniques. During the procedure, gentle and delicate movements are crucial to minimize the risk of iatrogenic injuries and postoperative complications.

### The birth of the seminal vesiculoscopy technique and its surgical approach

Ozgok et al. [23] first reported successfully removing seminal vesicle stones using transurethral endoscopy in 2005. The procedure involved placing the patient in the lithotomy position and inserting a 6.9F flexible ureteroscope through a guide wire to access the seminal vesicle. The stones were then crushed using forceps. Subsequently, Cuda et al. and Modi [21, 28] announced the clinical application of seminal vesiculoscopy lithotripsy, with both achieving favorable outcomes and contributing to developing the transurethral seminal vesiculoscopy technique. However, there is limited research comparing the efficacy of different surgical approaches for seminal vesiculoscopy. Chen et al. [29] reported that among 419 patients, methods A, D, and C were used in treatings eight (1.9%), 32 (7.6%), and 341 (81.4%) patients, respectively, while 38 cases (9.1%) resulted in failed operations. Liao et al. [5] included 305 patients and implemented methods A–C in seven (2.3%), 229 (75.1%), and 38 (12.5%) patients, respectively. The remaining nine cases (3.0%) resulted in failed operations. Besides, Hu et al. [30] conducted seminal vesiculoscopy in 38 hematospermia patients, with method A accounting for 3.21% and method C accounting for 88.89%. These findings highlighted that a smaller proportion of patients underwent successful seminal vesiculoscopy examination and treatment through natural orifices, with the majority being treated successfully by establishing a side channel at the bottom of the PU. However, a few patients experienced operation failure due to seminal vesicle gland atrophy, deformities, or other reasons [31]. It is worth noting that these cases were performed when the natural orifices of the ejaculatory duct could not be accessed, and there is a lack of comparative data regarding the postoperative effects of different approaches. However, the natural orifices approach remains the preferred method, and efforts should be made to minimize incisions and local invasive procedures. Our conclusion supports the notion that the recurrence rate of patients following the natural orifices approach is the lowest. Now analyzing the reasons behind our findings: (1) when considering the severity of the disease, the methods of fenestration in the PU and transurethral resection to expose the ejaculatory duct orifice are suitable for cases involving ejaculatory duct obstruction or spermary duct disease. These approaches are employed when natural orifices cannot be accessed normally. Pathological changes in the seminal tract at different stages are more pronounced in patients requiring these alternative methods than those who can undergo procedures through natural orifices. It is speculated that through natural orifices without incisions, the involvement of ejaculatory duct orifices in the seminal tract or vesicle pathology is less severe. Consequently, removing factors like seminal tract stones/cysts significantly reduces the recurrence rate of hematospermia. (2) the intraoperative effects on efficacy are noteworthy. Surgical procedures that can be performed through natural orifices without incisions preserve the original anatomical structure to a greater extent, minimizing interference with the entire seminal tract. This approach can attenuate the pressurization effect of seminal tract smooth muscle contraction on the seminal vesicles during ejaculation, counteracting or inhibiting reverse conduction force and preventing reinvasion of the seminal vesicles. Conversely, due to structural changes resulting from transurethral resection (TURED/TUIED) and fenestration in the PU, the fluid mechanics of the seminal tract may be altered, potentially exerting adverse effects on the seminal tract or vesicles. This phenomenon can be likened to the formation of turbulence when blood flows through areas of arterial dissection, which may facilitate thrombus formation. Furthermore, it alters the originally smooth and straight anatomical structure of the ejaculatory duct, making it susceptible to pressure impact at its end corners, thereby altering semen fluid mechanics. Local incisions or the creation of new channels can result in the formation of edema zones, scars, chronic inflammation, and potential narrowing of the seminal tract. These changes increase the risk of bleeding due to the recurrence of related lesions.

### Influence of anatomical factors on seminal vesicles endoscopy approach

It is worth noting that our data lacks a comparison involving anatomy-related influencing factors. Domestically and internationally, researchers are still in the exploratory phase of understanding the ejaculatory duct anatomy. The distribution of the ejaculatory duct orifice can directly impact the design of surgical approaches. Li et al. [32] were the first to propose that the ejaculatory duct opening is concealed due to the surface being covered with a one-way valve. This makes locating the ejaculatory duct orifices challenging. Shao et al. [33] conducted a statistical analysis of the ejaculatory duct orifices in 56 patients who underwent seminal vesicle surgery. They sectioned the edge of the PU from the top of the midline of the seminal verumontanum and classified the ejaculatory duct orifice into three types. The success rate of different access channels varies depending on the type.

Through the analysis of predictive factors, it has been determined that the presence of seminal tract stones/cysts is a reliable predictor of non-recurrence after hematospermia surgery using the natural orifices approach. Analyzing the reasons behind this predictive factor: (1) Many cases of seminal vesicle stones are associated with urinary tract infections, abnormalities, obstructions, and ejaculatory reflux [34]. Liu et al. [10] conducted seminal vesiculoscopy on patients with persistent and recurrent hematospermia and discovered that infections might initially stimulate the thickening of the ejaculatory duct membranous wall, resulting in stenosis and further exacerbating the infection due to impaired drainage. In some cases, infection and stenosis can contribute to stone formation. Conversely, stones can cause obstruction and frequent recurrence of infections. Briefly, stenosis, infection, and stones create a vicious cycle that perpetuates the infection, rendering it unresponsive to antibiotic treatment and ultimately leading to persistent or recurrent hematospermia [10]. Removing the stones breaks this vicious cycle, restoring the patency of the seminal tract and resolving the issue. (2) Urine reflux may also be the cause of the formation of stones in the seminal tract. When the discharge of ejaculatory duct secretions blocked, urine reflux, siltation and subsequent debris, coupled with urinary tract infection and seminal vesiculitis, can lead to the occurrence and formation of seminal vesicle stones and ejaculatory duct stones [3, 17, 21, 23, 28, 35–38]. The presence of these stones can create a one-way valve effect at the entrance of the seminal vesicles and ejaculatory ducts. By utilizing the natural orifice approach for stone crushing and removal, the obstruction caused by the stones can be relieved and the spermatic tract can be directly cleared. This approach reduces the likelihood of bleeding due to increased pressure in the seminal vesicles or ejaculatory ducts at a later stage.

In some clinical cases, urine reflux has been observed after transurethral ejaculatory duct related operation [21, 39], which could be attributed to accessing the ejaculatory duct through an unnatural orifice. Unlike the presence of sphincter function or peristalsis, the prevention of urine reflux in the ejaculatory duct relies solely on the acute angle formed between the ejaculatory duct and the PU. Animal experiments have demonstrated a similarity in the relationship between the seminal vesicle and the ejaculatory duct and that of the bladder and the urethra. When creating an opening to access the ejaculatory duct, it essentially forms a pseudo channel. If the opening size is too large, reflux may occur, whereas if it is too small, it may cause recurrent stenosis or even occlusion post-surgery [7, 35, 40, 41]. These two outcomes can contribute to the recurrence of hematospermia.

(3) Studies have indicated that stone formation can occur due to cyst blockage or impaired drainage of secretions during ejaculation [35]. The persistence of cysts and stones causes persistent blockage of the drainage. Moreover, patients with seminal vesicle stones often exhibit wall thickening in the seminal vesicle glands and ejaculatory ducts, further obstructing the discharge of secretions [12, 17, 35, 42, 43]. Therefore, the key to alleviating or resolving this issue lies in removing the underlying cause of the stones, which subsequently improves the condition of hypertensive seminal vesicle glands. By addressing these factors, post-operative hematospermia can be prevented.

Besides, we speculated that disturbances in the physiological state of the seminal vesicle glands may cause stone formation, resulting in hematospermia. While the seminal vesicle glands contribute to the production and excretion of semen containing mature sperm in coordination with other reproductive organs [2, 44], it is worth noting that the seminal vesicle itself is not a reservoir for semen. Its primary function lies in the secretion of a liquid comprising proteins, fructose, and various enzymes that nourish sperm [2, 45]. These components indirectly contribute to the stone composition. When the smooth discharge of semen is impeded, the concentration of these substances increases, thereby accelerating stone formation.

This study has several limitations, primarily due to its retrospective nature, which inherently introduces bias. Although we have conducted a comparative analysis of the efficacy of each influencing factor and evaluated the differences in surgical approaches, it is important to acknowledge the need for future investigations with larger case numbers and more comprehensive research and statistical analyses on the technical aspects. These endeavors are expected to yield clearer surgical indications for treating hematospermia using seminal vesiculoscopy, further enhancing surgical efficacy and uncovering additional insights that can guide the widespread application of seminal vesiculoscopy, thereby better-addressing patient concerns.

## Conclusion

The Transurethral seminal vesiculoscopy technique demonstrates a low postoperative recurrence rate in treating hematospermia. Among the various approaches, the intraoperative use of natural orifices through the ejaculatory duct exhibits the lowest recurrence rate. Additionally, seminal tract stones/cysts effectively predict favorable postoperative outcomes.

## Data Availability

The datasets used and/or analysed during the current study available from the corresponding author on reasonable request. The images in study were appropriated permission by patients.

## Abbreviations

TURED/TUIED: Assisted transurethral resection/incision of ejaculatory ducts
PU: Prostatic utricle
MRI: Magnetic Resonance Imaging
CT: Computerized Tomography
TRUS: Transrectal ultrasound screening
